# Making Transmission Models Accessible to End-Users: The Example of TRANSFIL

**DOI:** 10.1371/journal.pntd.0005206

**Published:** 2017-02-02

**Authors:** Michael A. Irvine, T. Deirdre Hollingsworth

**Affiliations:** 1 Zeeman Institute, University of Warwick, Coventry, United Kingdom; 2 School of Life Sciences, University of Warwick, Coventry, United Kingdom; Imperial College London, UNITED KINGDOM

## User Interfaces to Models

A recent review of providing results for public health policy stated that "to provide an interactive interface… should be very easy to do for any model" and encouraged modellers to provide such interfaces [1]. However, whilst the technologies for such interfaces have been around for many years, there are remarkably few examples of such interfaces available to researchers [2]. There has been an increased practice of releasing model code so that other experienced modellers can use it, as highlighted by the neglected tropical disease (NTD) modelling consortium papers in 2015 [3]. Developing more user-friendly interfaces to complex transmission models faces several challenges [4]:

- Access—for users with limited modelling expertise.
- Speed—analyses produced quickly without expensive computer resources.
- Characterisation of uncertainty—usually through repeated runs of the model, resulting in a higher processing burden.
- Ease of use—requires design choices, including instructive inputs.
- Clarity of presentation—limiting misunderstanding of the model and its outputs.
- Responsiveness to needs—flexibility to iteratively update the interface through a consultation with intended end-users.
- Range of users—different users have different needs, and it is challenging to survey and understand all of these needs.

Here, we introduce a newly developed online web interface for the lymphatic filariasis transmission model TRANSFIL, which is validated for *Wucheria bancrofti* transmission [5] and has been used in a recent study of the potential impact of the new triple drug [6]. We hope this demonstrates how modern web technologies can be exploited to produce model interfaces that are able to overcome the challenges listed above, although some challenges remain (Box 1). This tool is targeted at users who have some awareness of and an interest in mathematical modelling for lymphatic filariasis policy and who would like to investigate the models further, but who do not have the technical expertise to program the models themselves. This will include some researchers and policy makers in the area of NTDs. We hope it will generate an active discussion between the modellers and these users on what types of analyses are most useful for these users.

**Box 1. Advantages and Disadvantages of a Web Interface to the Model**

**Advantages**
1. Access to the model for nonexpert users.
2. Real-time results for users using local processing.
3. Interactive input and output focused on disease-specific goals.

**Disadvantages**
1. Potential misinterpretation or misuse of results due to lack of expert guidance; for example, the dynamics of breaking transmission are likely to be highly locally specific and the modeling results should considered in this context.
2. Limited parameters can be changed in the model. End-user doesn’t have full access to model through interface.
3. Limited tailoring to local settings.

## Lymphatic Filariasis

Lymphatic filariasis is a parasitic, mosquito-borne infection currently targeted by the World Health Organization (WHO) for elimination as a public health problem by 2020 [7]. The current recommended strategy for treating lymphatic filariasis is to provide five rounds of mass drug administration (MDA) with at least 65% coverage for five years and, then, surveys to evaluate whether a threshold of <1% microfilaraemia or <2% antigenaemia has been followed by retesting to evaluate where transmission is continuing [8]. There are currently multiple drug regimens in use by programmes in addition to vector control. Therefore, this disease system is ideal to demonstrate intervention complexity through a modelling interface.

## Aims of the Interface

The aims of the interface are to provide a user-friendly, nontechnical way of producing modelling results customized for the end-user. It should readily produce analyses on these customized scenarios and provide tools for comparison and probing of the scenarios. Whilst there have been a number of modelling results discussing the importance of coverage, compliance, treatment strategy (yearly and twice yearly), the role of vector control, and baseline prevalence on achieving the 2020 goals (e.g., [5]), readers have not yet been able to investigate these interactions for themselves. The primary users of the interface are expected to be researchers and strategic policy makers who are investigating the impact of different treatment strategies to achieve the 2020 goals. Therefore, alongside model simulations, we provide box plots comparing user-defined scenarios in terms of the number of rounds to achieve <1% microfilaraemia prevalence. These scenarios are defined in terms of baseline prevalence, coverage and patterns of adherence, and vector control.

It should be noted that the model is focused on standard MDA using the approved WHO guidelines, for example, excluding areas where loaisis is coendemic [9]. The interface is not designed to be a decision tool for detailed local policy planning, which would also require more tailoring and fitting of the model to local data, thus making it difficult to automate.

## Overview of the Interface

**Fig 1:**
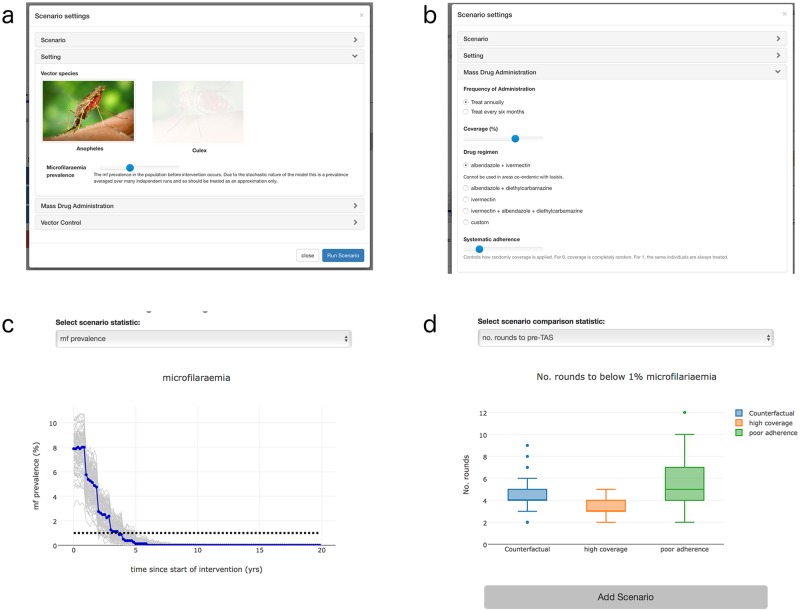
Overview of modelling tool. (a) Selection panel used to design scenario. Dominant vector species can be selected and baseline prevalence is controlled using a slider. (b) Second selection panel. The frequency, coverage, drug regimen, and systematic non-adherence can all be altered. Information on specific regimens also appears when one is selected. (c) Output results for a scenario. Median value shown in blue, with separate runs in grey. Other outputs can be selected. (d) Scenario comparison outputs. Number of rounds to halting MDA (pre-TAS) as well as other outputs can be selected.

The model and interface were developed as a JavaScript application. This means it will run in any modern web browser, operating system, and on a range of machines, including mobile devices. The interface is accessed via a URL and results are stored locally and can also be downloaded to the user’s computer (Fig 1).

## Technical Advancements

By exploiting modern web technologies, including JavaScript, HTML5, and CSS3, the model and interface are able to run on an individual’s machine without the need to constantly access resources from an external server or database. Modern web browser JavaScript engines use just-in-time compilation and an array of optimizations, making it a viable option for simulating complex epidemic models.

Slider controls were used for the vast majority of user input (Fig 1a and 1b). This means that the designer can set the range and step size for a parameter and prevent the user from incorrectly inputting an extreme parameter, which may lead to spurious results (e.g., having a negative number for a positive rate, which may lead to the model running without error, but would produce results that aren’t meaningful).

Many free-to-use open source libraries exist currently for JavaScript. Here, plotly.js and d3.js were used for the graph plotting, as well as the web framework bootstrap for the user interface [10, 11]. These are well-established libraries used for enterprise software and are under continual development cycles, meaning they are quick to run and should conceivably work well into the future.

## Future Developments

The approach of running and displaying modelling results as a web application can be applied to other transmission models of NTDs. The approach would also be amenable for users to upload data and run analyses on these to further calibrate the modelling results, requiring technical advances in rapid model fitting. There is also an important technical challenge in selecting simulations that start exactly at, say, 10% prevalence at the time the interventions begin, because of the stochastic nature of the model. The model settings and outputs could also be adapted to take into account more specific program needs, such as populations at risk, the relationship between true and reported coverage, financial resources, and other logistic demands. The approach could also be developed to share results and data between researchers and public health workers using the web interface as a portal to achieve this.

## Summary

This represents an approach to using established web technologies to develop a modelling tool accessible to a wide range of individuals. The benefits of this approach include the ability to quickly generate results, the ability to change key assumptions in the modelling, and the lack of need to install software.

## Link

The modelling tool can be accessed through a web browser at the following URL: http://www.ntdmodelling.org/transfil
